# Editorial: Robotics to Understand Animal Behaviour

**DOI:** 10.3389/frobt.2022.963416

**Published:** 2022-07-11

**Authors:** Liang Li, Sridhar Ravi, Chen Wang

**Affiliations:** ^1^ Department of Collective Behaviour, Max Planck Institute of Animal Behavior, Konstanz, Germany; ^2^ Centre for the Advanced Study of Collective Behaviour, University of Konstanz, Konstanz, Germany; ^3^ Department of Biology, University of Konstanz, Konstanz, Germany; ^4^ School of Engineering and Information Technology, University of New South Wales, Canberra, NSW, Australia; ^5^ The National Engineering Research Center of Software Engineering, Peking University, Beijing, China; ^6^ The State Key Laboratory of Turbulence and Complex Systems, Intelligent Biomimetic Design Lab, College of Engineering, Peking University, Beijing, China

**Keywords:** bioinspired robot, animal behaivor, collective behaivor, hydrodynamic interaction, robots in biology

For centuries, designers and engineers have turned to biological systems to seek inspiration for constructing functional, flexible, and robust robots (Bar-Cohen, 2005), which has matured into a significant branch of robotics. Recently, biologists and interdisciplinary teams are using robots as viable testbeds to help explore and evaluate hypotheses in animal behaviour (Webb, 2000; Marras and Porfiri, 2012; Gravish and Lauder, 2018; Landgraf et al., 2021). This strategy offers a suitable alternative to traditional biological methods, which have severe limitations including the feasibility to minimize confounding influences and isolate effects on living specimens, the effects of the experiment and the experimenter on the study animal, and other limitations imposed by the behaviour of the animals. For example, a fish-like robot can greatly help biologists collect *in-situ* data on fish schooling behaviour with limited interferences from humans or other factors (Katzschmann et al., 2018), explore potential mechanisms of hydrodynamic benefits among individuals (Li et al., 2020), and test hypotheses in collective motion by including the robot in the loop of collective animal behaviour (Bonnet et al., 2018). The goal of the research topic is to showcase those interdisciplinary studies that reverse the idea of bio-inspired robots by applying engineering methods (theory, simulation, or experiment) to study animal behaviour.

After the peer review process, this topic accepted 5 articles, including 4 research articles and 1 perspective article. Schwab et al. built a biorobotic fish as an experimental model and systematically explored subcarangiform fish swimming behavior. Equipped with gallium-indium (eGaIn) sensors the robot could sense proprioceptive signals like a live system. Detailed flow fields collected by PIV (Particle image velocimetry) were further explored with nascent analytical techniques such as DMD (Dynamic mode decomposition) thus overcoming the significant challenges of performing similar experiments on real fish. In another study, Li et al. applied CFD (computational fluid dynamics) to study how fish decode information about their neighbor’s swimming via near flow fields. They simulated the detailed flow fields around schooling fish and found FFT (Fast flourier transform) was able to extract the relative position, phase differences, and the tail-beat frequency of its neighbor, thus posing a novel hypothesis about the potential sources of information to real fish. Stefanec et al. constructed a small group of robots to interact with the queen bee in a colony. They justified the effectiveness of these robots via “queen court events” (QCE)—where the queen is surrounded by the court worker bees while resting. Olejnik et al. applied both CFD and an experimental micro-air-vehicle platform to understand how insects achieve incredible agility and cope with wind unsteadiness by utilizing passive and/or active flight control strategies. Through experiments and simulation, they found flying flies are partially passive stable in lateral winds due to their asymmetry wing-beat Horsevad et al. made an argument that as a complement to the simulation studies, experiments with robots can be a powerful tool to understand collective behavior.

Taken together, the studies presented in this special issue highlight the benefits robotic platforms offer interdisciplinary teams seeking insights into animal behavior and hopefully further inspire the cross-pollination of knowledge and expertise across organismal/behavioral biology and robotics.
